# Superchiral Light Emerging from Bound States in the Continuum in Metasurfaces of Si Nanorod Dimers

**DOI:** 10.1021/acsphotonics.4c00938

**Published:** 2024-09-03

**Authors:** Jose Luis Pura, Beatriz Castillo López de Larrinzar, Minpeng Liang, Antonio García-Martín, Jaime Gómez Rivas, José A. Sánchez-Gil

**Affiliations:** † Instituto de Estructura de la Materia (IEM), Consejo Superior de Investigaciones Científicas, Serrano 121, 28006 Madrid, Spain; ‡ GdS-Optronlab, Física de la Materia Condensada, Universidad de Valladolid, Paseo de Belén 19, 47011 Valladolid, Spain; ¶ Instituto de Micro y Nanotecnología IMN-CNM, CSIC, CEI UAM+CSIC, Isaac Newton 8, Tres Cantos 28760 Madrid, Spain; § Department of Applied Physics and Science Education and Eindhoven Hendrik Casimir Institute, Eindhoven University of Technology, P.O. Box 513, 5600 MB Eindhoven, The Netherlands; ∥ Institute for Complex Molecular Systems-ICMS, Eindhoven University of Technology, P.O. Box 513, 5612 AJ Eindhoven, The Netherlands

**Keywords:** Superchiral light, bound states in the continuum, chirality, helicity, near-field

## Abstract

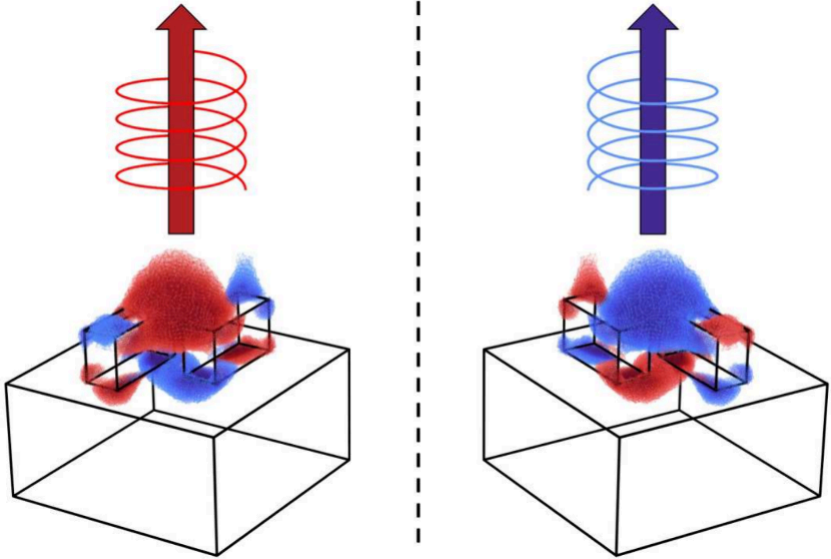

Bound states in the continuum (BICs) in all-dielectric metasurfaces enhance light–matter interaction at the nanoscale due to their infinite *Q* factors and strong field confinement. Among a variety of phenomena already reported, their impact on chiral light has recently attracted great interest. Here we investigate the emergence of intrinsic and extrinsic optical chirality associated with the excitation of BICs in various metasurfaces made of Si nanorod dimers on a quartz substrate, comparing three cases: parallel nanorods (neutral) and shifted and slanted dimers, with/without index-matching superstrate. We analyze both the circular dichroism (CD) of the far-field (FF) interaction and the helicity of the near-field (NF) distribution. We show that the best approach to achieve chiral response in the FF based on extrinsic chirality is to exploit quasi-BICs (q-BICs) appearing in the case of slanted nanorod dimers. By contrast, the helicity density is largely enhanced in the case of shifted dimers, as it presents intrinsic chirality, with values 2 orders of magnitude larger than those of circularly polarized plane waves. These so-called superchiral electromagnetic fields concentrated at the nanoscale within the metasurface hold promise of appealing implications in phenomena such as strong-coupling, photoluminescence emission, or other local light–matter interactions.

## 1 Introduction

Bound-states in the continuum (BICs) are attracting a great deal of attention in photonics due to their inherently high (ideally infinite) Q factors, despite being resonant modes lying in the continuum.^1,2^ Planar (nondiffractive) arrays of particles, i.e. metasurfaces, favor the emergence of such BICs due to the limitations they impose upon the available radiation channels.^3−6^ In addition, such platforms hold potential as planar photonic devices, stemming from the large enhancement of light-matter interaction processes that the supported high-*Q* BICs, or quasi-BICs (q-BICs), convey, such as lasing,^7,8^ exciton-polariton condensation,^9^ sensing,^10^ and a variety of other nonlinear processes.^11^

Any object that cannot be superimposed with its mirror image possesses intrinsic chirality. Chirality can be present in both matter, i.e. physical structures, and also light itself can present polarization states with opposite handedness, right and left. In chemistry, chiral light has been instrumental in elucidating molecular structures and dynamics offering insights into fields such as biomolecular interactions or drug development.^12,13^ In optics and photonics, chiral light enables advanced manipulation of light-matter interactions, leading to innovations in optical trapping, or chiral sensing.^14−16^ In emerging fields like quantum optics and quantum information processing, chiral light plays a crucial role in generating and manipulating entangled states, enabling advancements in quantum communication and computing.^16−18^ It has been recently shown that BICs may enhance the chiral response of metasurfaces,^19,20^ and also chiral emission through chiral q-BICs can be achieved.^21−23^ A wealth of configurations have been proposed supporting BICs or q-BICs that claim various intrinsic and extrinsic chiral processes.^24,25^ Intrinsic chiral BICs can be obtained by either achieving BICs in a metasurface (MS) based on chiral meta-atoms,^26^ or leveraging the out-of-plane dimension to break the symmetry in the direction of propagation of the electromagnetic fields, thus generating chirality.^21,27−29^ Furthermore, it is also relatively easy to obtain extrinsic chiral q-BICs (chiral at an oblique angle) by only breaking the in-plane symmetries^30−32^ extending the applications of chiral q-BICs.^19,25,33^

Superchiral light is defined as an electromagnetic field whose chirality (helicity density) is higher than that of free-standing monochromatic circularly polarized light (CPL). The first approaches to obtain such fields considered a CPL reflected on a mirror with reflectivity *R* < 1, the superposition of the incident and reflected waves result in a total field with a chirality exceeding that of CPL.^34,35^ Further development showed the possibility of obtaining superchiral fields in the vicinity of achiral^36,37^ and chiral nanostructures,^38−40^ also showing an enhancement of the resulting circular dichroism (CD).^35,41^ More recent works also found superchiral fields and CD enhancement on metasurfaces based on nanodisks^42,43^ or nanoholes,^44^ with very recent applications on virus sensing.^45^

We present in this manuscript a study of the chiral properties emerging from bound states in the continuum in metasurfaces of poly-Si rod dimers. Three different cases are presented corresponding to the three symmetries that support the presence of BICs/q-BICs and the metasurfaces are studied both from the far-field and near-field approaches. The results show the possibility of obtaining extremely superchiral near-field distributions while preserving the inherently high values of the Q factor of BICs and q-BICs. The combination of superchiral fields and large Q factors foresees exciting applications to enhance the interaction between chiral light and chiral matter, like chiral strong-coupling, or chiral lasing.

## 2 Methodology and System Description

The studied systems are based on arrays of dimers of poly-Si rods. The MSs are studied by two complementary approaches: quasi-analytic calculation with a coupled electric-magnetic dipole (CEMD) model, and full numerical calculations through COMSOL Multiphysics. The CEMD model provides the response of the system within the dipolar approximation by considering the electric and magnetic polarizability tensors of the individual rods, α̃_e_ and α̃_m_. Further details about the CEMD formalism can be found elsewhere.^46,47^ The main advantage of the CEMD model is the much faster calculation, compared to numerical models. On the other hand, the numerical solution provides the complete response of the system (beyond the dipolar approximation) according to Maxwell’s equations, but at the expense of a much longer computational time.

**Figure 1:**
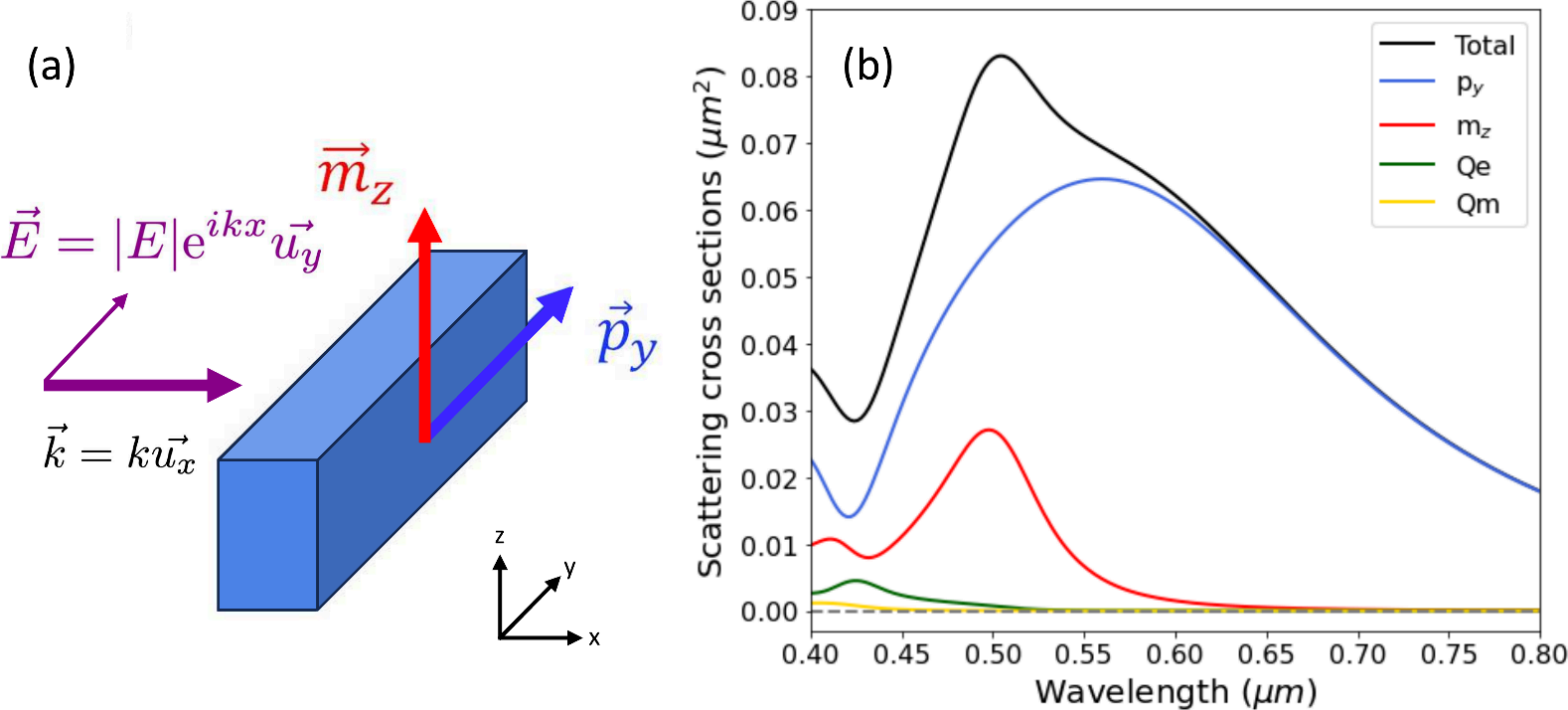
(a) Scheme of the main electric and magnetic dipolar contributions of the isolated rod when it is illuminated with a linear electromagnetic wave impinging in the *x*-axis and polarized in the *y*-axis. The dimensions of the rod are 60 × 160 × 90 nm^3^. (b) Scattering cross-section and its multipolar decomposition of a single poly-Si rod in a homogeneous medium with *n* = 1.46.

In this work, the polarizability tensor is obtained after solving numerically the full vectorial problem of an isolated poly-Si nanorod using Finite-Difference Time-Domain (FDTD) techniques,^48^ hence the quasi-analytic tag. Dipolar and quadrupolar contributions are then extracted from the numerical values of the current density following the procedure reported in ref (49). It is worth to be noted that this procedure has been recently used to get also the dressed dipolar contributions to the scattering cross sections in nanostructures consisting in disconnected, highly interacting subunits.^50,51^ In Figure 1, we present the main multipolar contributions for a single poly-Si nanorod (using the experimental values of the dielectric function of polycrystalline silicon as measured in ref (52)), fully embedded in a refractive index of n = 1.46, under incidence along the x̂-direction and polarization across the ŷ-direction in the form of their contributions to the scattering cross section. As the system is certainly anisotropic, the cross section, and then the polarizabilites, depend on incidence and polarization state. The corresponding polarizabilities used by the CEMD in this work are provided in the SI. These are the ones required to obtain the full electric and magnetic polarizability tensors and account for the strong in-plane interactions occurring within the nanorod dimer array. In the spectral region of interest, the strongest, lowest-order component is the longitudinal (*p*_*y*_) electric dipole (ED), with a resonance at λ ∼ 560 nm. Other weaker ED contributions have to be taken into account, being resonant at λ ∼ 505, 475 nm. In addition, there is a strong magnetic dipole (MD) contribution (*m*_*x*_), peaking at λ ∼ 560 nm; and weaker ones at wavelengths similar to the weaker EDs λ ∼ 505, 475 nm. Quadrupolar terms appear at higher energies for λ < 500 nm, beyond the spectral region we are focused on. This analysis supports the use of coupled-dipole approximations, and yields in turn accurate dipolar contributions to the polarizability tensor of individual nanorods. Among the variety of coupled-dipole formulations including electric and magnetic contributions,^53,54^ we will use below the CEMD described in Abujetas et al.^46^ Recall that, for the CEMD to be valid, apart from the condition just mentioned, the nanorods should be sufficiently separated from each other, both in the dimer within each unit cell and between adjacent unit cells, so that the near-field interaction among nanorods is reasonably accounted for within the dipolar approach. It should be emphasized that CEMD models provide meaningful results with deep physical insight, even quantitatively accurate in many instances, being in turn an order of magnitude (at least) faster than typical full numerical simulations.

**Figure 2:**
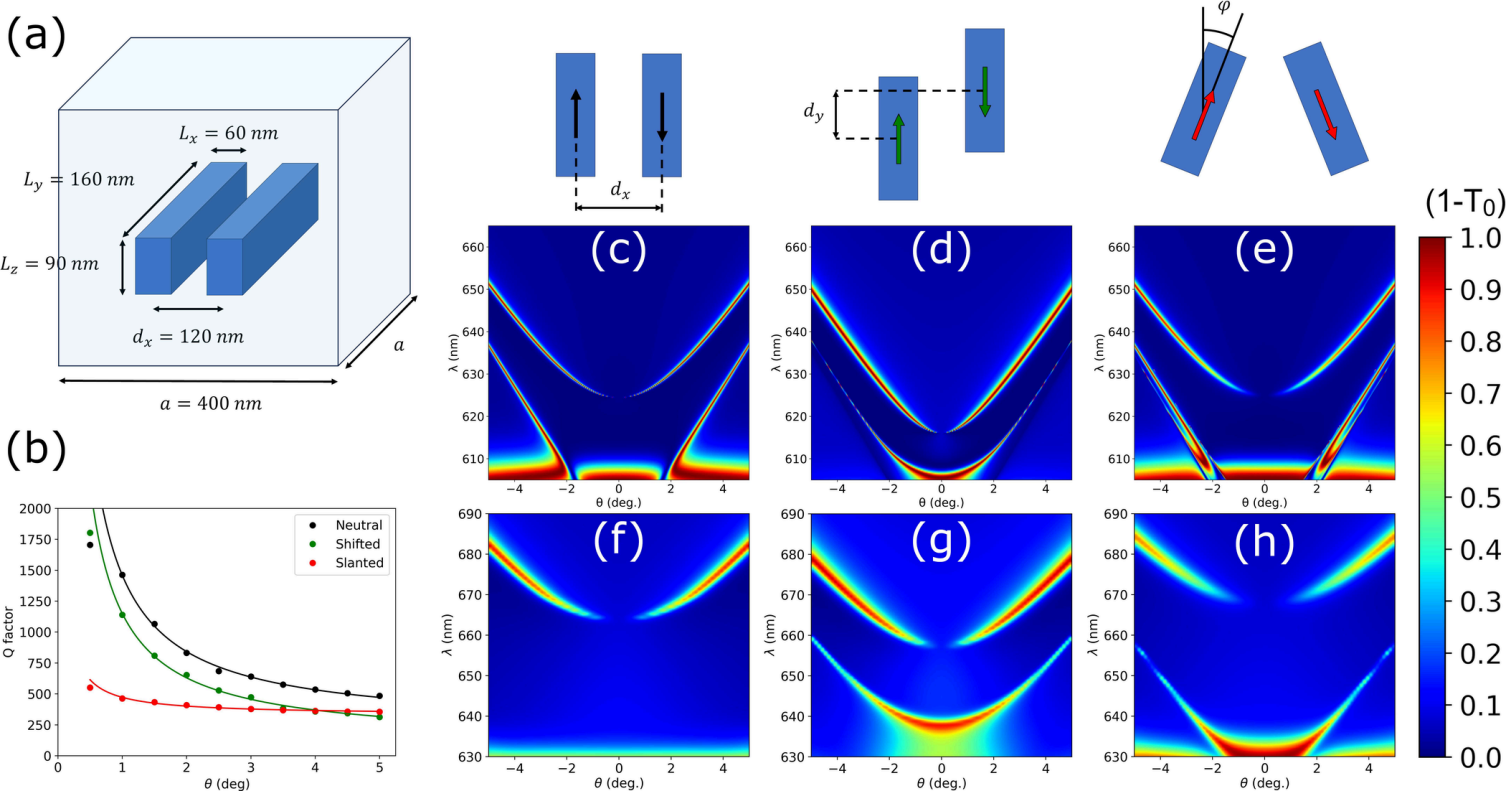
(a) Scheme of the unit cell of the studied metasurface with all the relevant dimensions: *a* = 400 nm, *L*_*x*_ = 60 nm, *L*_*y*_ = 160 nm, *L*_*z*_ = 90 nm, *d*_*x*_ = 120 nm, *n* = 1.46. (b) *Q*-factors of the BIC/q-BIC bands extracted from the COMSOL extinction spectra. The continuous line reflects the expected dependence on the angle as 1/sin θ. (c–e) Extinction (1 – *T*_0_) bands calculated with CEMD for the three studied cases: neutral, shifted (*d*_*y*_ = 120 nm), and slanted (φ = 10°), for the TE polarization. The CEMD calculations have been carried out including the contributions from all the electric and magnetic dipolar polarizabilities. (f–h) The same as panels c–e but calculated with COMSOL Multiphysics, with the wavelength scale slightly shifted to facilitate the comparison with the CEMD bands. On top of the extinction spectra, the schemes of the relative orientation of the two electric dipoles involved in the *p*_*y*_ BIC and q-BIC under study are shown.

Once the electromagnetic response of the individual rods is characterized we proceed to the study of the MSs. The considered systems comprise pairs of rods of polycrystalline Si placed on a square array as depicted in Figure 2a. The rods are deposited over a quartz substrate (n = 1.46) and the environment is set to two different scenarios: first, an environment with the same refractive index, resembling a typical index-matching scenario; second, a superstrate with a higher n = 1.6, inducing an index mismatch across the interface that enhances certain chiral effects, as will be shown below. Note that the chosen refractive index corresponds to that of a typical active material (PMMA with a dye) coating the MS. The dimensions of the rods are tuned so that the electric dipolar resonance on the ŷ-direction (*p*_*y*_) of each rod lies close to the 600 nm region, which is relevant for visible range applications, while remaining suitable for its manufacture with typical e-beam lithographic techniques at the same time. This results in the values *L*_*x*_ = 60 nm, *L*_*y*_ = 160 nm, and *L*_*z*_ = 90 nm. The rod distance (center-to-center) *d*_*x*_ = 120 nm, is large enough to analyze the two rods within the dipolar approximation and to allow lithography. Finally, the lattice parameter is fixed to *a* = 400 nm. From this initial configuration of maximum symmetry (referred to as neutral), we also study two variations to ascertain their optical activity: the first one (shifted) introduces an opposite vertical shift of both rods, *d*_*y*_, which is set to 100 nm if not stated otherwise; the second one consists on an antisymmetric rotation around the *z*-axis (slanted), the rotation angle φ is set to 10°. The upper part of Figure 2 shows a scheme of these three configurations.

The results for the extinction bands under TE illumination (*y*-polarization) for the CEMD and COMSOL calculations are provided in Figures 2c-e and 2f-h, respectively. The bands correspond to the ΓX direction, which means that the incident angle, θ, is contained in the *xz* plane. There is a good agreement between the CEMD and COMSOL calculations in the nondiffractive regime, which manifests the validity of the dipolar approximation for this system. Nonetheless, note that there is a slight mismatch in the wavelength at which the BIC emerges, most likely due to higher-order multipoles arising in the near-field interaction between adjacent rods. To facilitate the comparison between both bands, this systematic shift is compensated by centering the wavelength scale in Figures 2c–h at the BIC frequency, while maintaining the wavelength range identical in all of them; in this manner, it is evidenced that the q-BIC dispersion is very similar in both kind of calculations.

We will be interested in the lowest energy mode, labeled *p*_*y*_, which corresponds to a pair of antisymmetric electric dipoles along the *y*-axis, as represented in the upper schematics in Figure 2. This mode becomes a symmetry-protected BIC at normal incidence for the neutral and shifted cases, as observed in the extinction bands. Indeed, it is important to note that, for the shifted case, the BIC nature of the mode is not disturbed by the vertical displacement of the rods as analytically shown in ref (46). For the slanted case, the mode is not a pure TE mode (*y*-polarized); rather, the rotation of the rods involves an *x*-component that results in a TM contribution overlapping with the TE band, which makes the mode hybrid, see Figure S2 in the Supporting Information. Thus, the mode can radiate at normal incidence, where the TM band presents a nonvanishing linewidth, making the mode a q-BIC with a finite Q factor. This underlying physical mechanism has also been described in a similar configuration with rod dimer metasurfaces.^28,55,56^ To confirm the nature of those BICs and q-BICs, we show in Figure 2b the Q factors of the q-BIC bands for all three cases as a function of the angle as extracted from the COMSOL extinction spectra in Figure 2f-h. In addition, a divergence dependence on the asymmetry parameter (1/sin θ in this configuration) is shown. It is evident that the Q-factors for the neutral and shifted cases diverge upon decreasing the angle of incidence, revealing these dark modes are BICs at the Γ-point; however, for the slanted case, the *Q*-factor saturates close to θ = 0°, indicating that the dark mode is a q-BIC with finite *Q*-factor even at the Γ-point.

The *p*_*y*_ nature of the lowest-order (quasi-)BIC band is verified by recalculating the extinction through the CEMD formulation including only the contribution from α_*e*,*y*_. As observed in S2, the BIC extinction bands are fully reproduced by the interaction of (only) the longitudinal (*y*-direction) ED of each nanorod within the CEMD.

In order to ascertain the optical activity of the *p*_*y*_ BIC, two different magnitudes are studied. First, the circular dichroism (CD) is considered for FF interaction. It is defined as the different response of the metasurface for the two circular polarizations: left-hand circularly polarized (LCP) and right-hand circularly polarized (RCP):
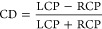
1

This magnitude can be calculated for either reflectance (R), transmittance (T), or extinction (1-T). The base for the states of circularly polarized light (CPL) can be defined at normal incidence as
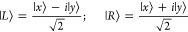
2When changing the angle of incidence the states are rotated accordingly to remain orthogonal to *k⃗*.

The other relevant magnitude for the analysis of the optical activity is the helicity density, which is defined as^41,42,57,58^
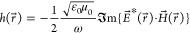
3

This magnitude can be calculated for any field distribution, however, since it is quadratic in the fields and it also depends on the frequency it is convenient to provide its normalized value for comparison. To do this we will normalize it with respect to the helicity density of a (left-handed) circularly polarized plane wave with the same frequency:
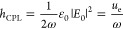
4where *u*_e_ is the energy density. With this normalization, a value of *h*_n_ = *h*/*h*_CPL_ = ±1 will correspond to an electromagnetic field with the same helicity as circular light (LCP or RCP), while a value greater than 1 is considered a superchiral field.^35^ There exist analogous definitions of the chirality (also referred to as optical chirality density, OCD^59,60^) which use the fields *D⃗* and *B⃗* instead of *E⃗* and *H⃗* obtaining similar conclusions. Many approaches also consider *E⃗* and *B⃗*,^34,61^ which is completely equivalent to eq 3 for nonmagnetic materials.

## 3 Results and Discussion

Once the system is defined, as well as the relevant BIC, we proceed to analyze the optical activity of the system as different symmetries are broken. The neutral case presents *C*_2_ symmetry with respect to the center of the unit cell, as well as two mirror symmetry planes: *zx* and *yz*. The shifted case breaks the two mirror symmetries while maintaining *C*_2_ symmetry. On the other hand, the slanted case breaks *C*_2_ and *zx* mirror symmetries while maintaining *yz* mirror symmetry. The third case (maintaining only *zx* mirror symmetry) could be obtained by rotating only one of the rods 90°; however, the obtained mode is not compatible with the BIC condition and becomes radiative for all angles.

### 3.1 Circular Dichroism and Far-Field

**Figure 3:**
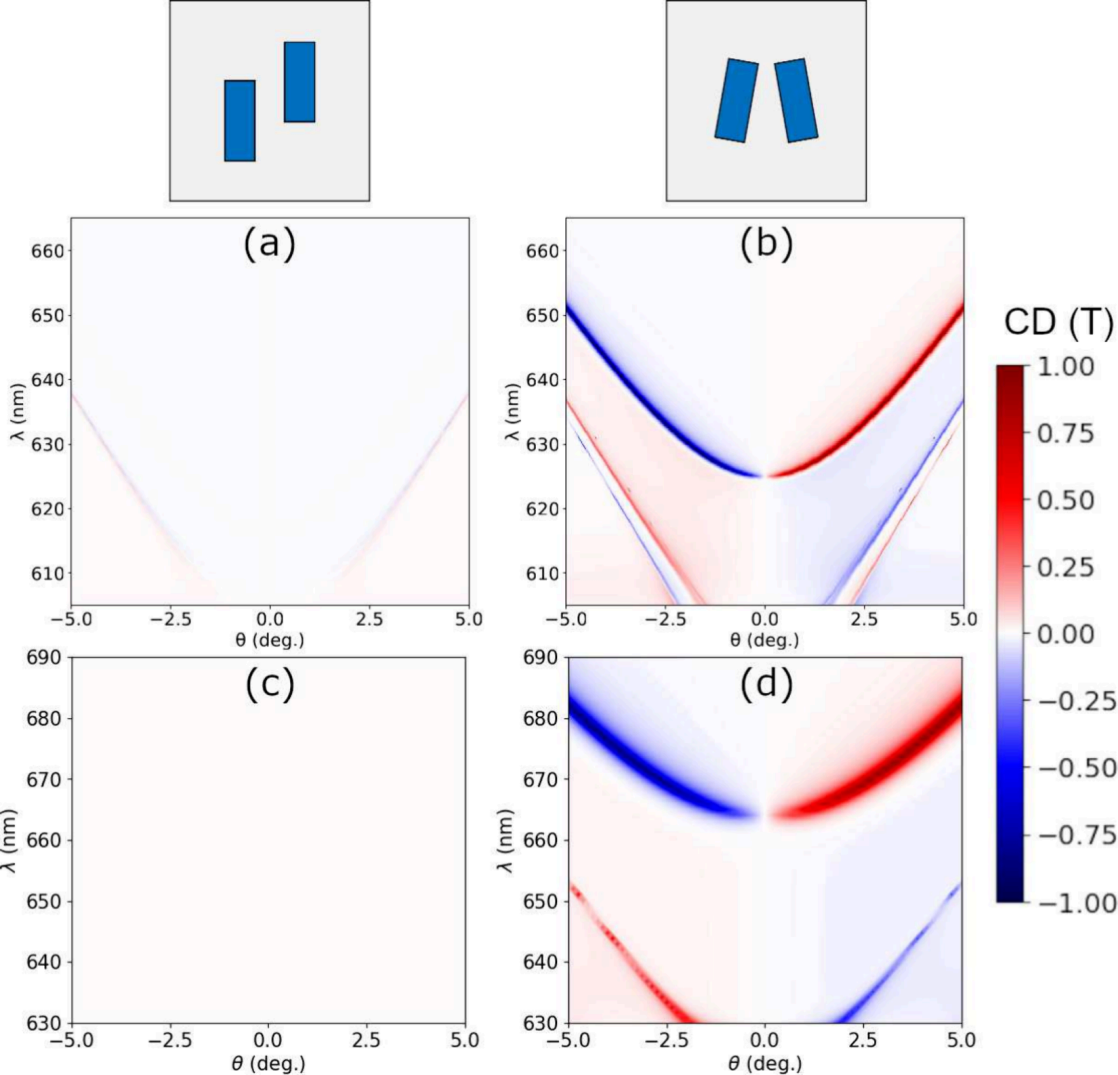
Circular dichroism in transmission, CD(T), calculated with the CEMD model (a, b) and COMSOL Multiphysics (c, d) for Si rod dimer metasurfaces with rod and lattice dimensions as in Figure 2, and for (a, c) shifted (*d*_*y*_ = 100 nm) and (b, d) slanted (φ = 10°) cases, respectively.

We start by studying the FF optical activity. Figure 3 presents the CD calculated both with the CEMD model (a, b) and COMSOL (c, d) for the shifted and slanted cases. The neutral case is not presented as its maximum symmetry results in the total absence of CD. In all cases, the CD is identically zero at normal incidence due to either *C*_2_ or *yz* mirror symmetries. In fact, any planar structure embedded in a homogeneous dielectric medium is not truly chiral because of reciprocity and the existence of an inherent mirror (*xy* plane) symmetry.^62−65^ For off-normal incidence, we can observe that the slanted case, Figures 3b and d, presents much higher values of CD than the shifted case, almost reaching unity. This can be easily understood as a result of the different symmetries. For the shifted case the two electric dipoles induced in the rods always point in the ŷ-direction as depicted in Figure 2b. Even when θ ≠ 0 the dipoles can only interact with the *y*-polarized component of the incident field (TE polarization) which rules out the possibility of a different response for RCP and LCP light. This is reflected in the complete absence of CD for the BIC, Figures 3a and c. For the slanted case, the rotated electric dipoles present *x* and *y* components of the polarizabilities but also the rotation includes nonzero *xy* and *yx* components.
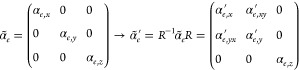
5Where *R* = *R*(φ) is the rotation matrix around the *z* axis with angle φ. The off-diagonal terms allow the hybridization of the states of the two dipoles allowing a different response for RCP and LCP light and resulting in the observed large values of extrinsic CD.^20^

### 3.2 Influence of the Substrate

In order to allow the presence of nonzero intrinsic local chirality it is mandatory to break the symmetry along the direction of propagation, i.e. the *z* axis. This can be easily done for MSs by using a material for the upper environment with different optical properties than the substrate. Moreover, this is the typical situation when doing luminescence or strong-coupling measurements on this kind of system.^9,66,67^ According to this, for all the NF calculations the substrate is maintained as quartz (*n*_sub_ = 1.46) while the superstrate is set to a constant refractive index of *n*_sup_ = 1.6. This configuration resembles the deposition of an active material over the MS.

**Figure 4:**
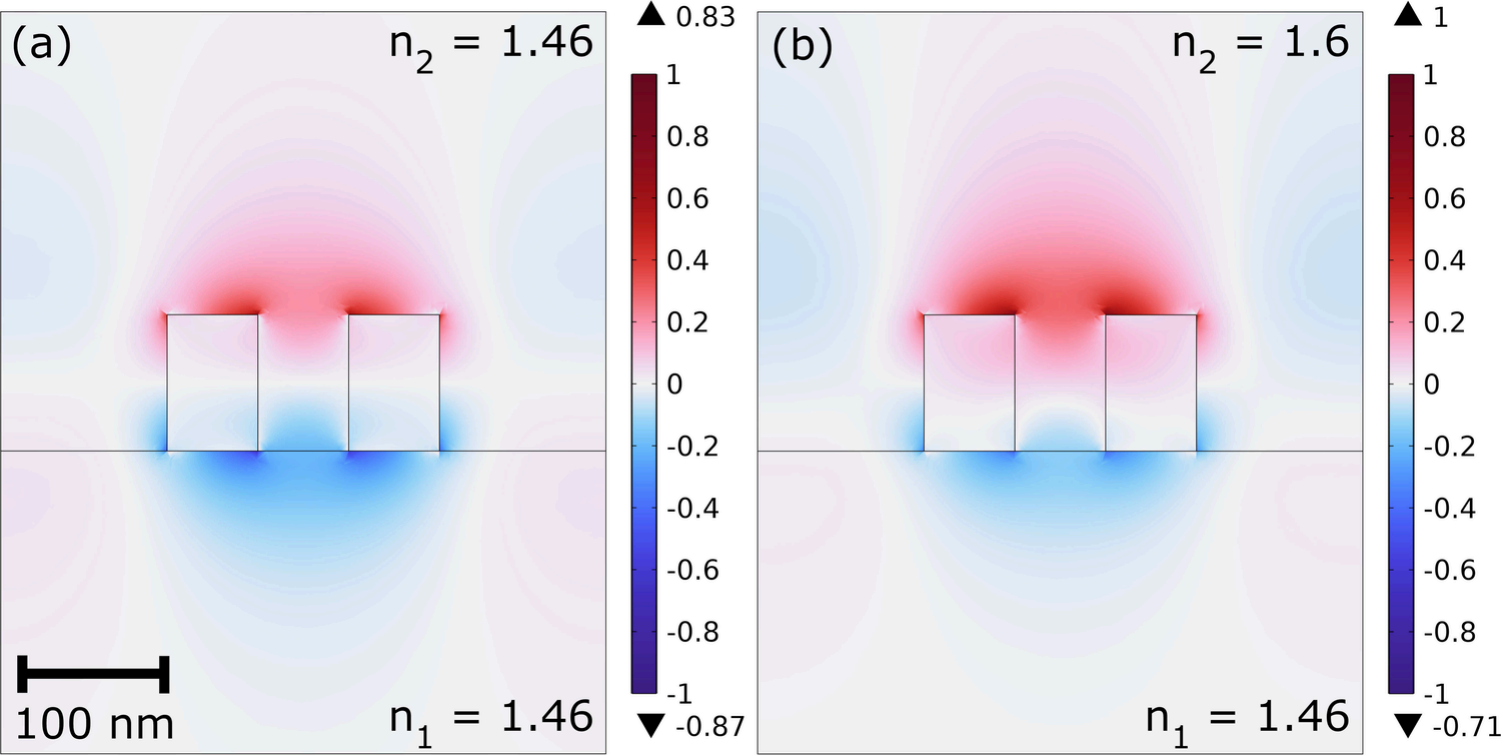
Helicity density for Si rod dimer metasurfaces with rod and lattice dimensions as in Figure 2 for the shifted case (*d*_*y*_ = 100 nm). The helicity density is normalized to the maximum value of both cases for direct comparison. (a) System under index matching conditions (*n*_1_ = *n*_2_ = 1.46). (b) Same as (a) but with a superstrate with a different refractive index (*n*_2_ = 1.6) deposited over the metasurface. The color bar top and bottom numbers display the maximum and minimum values of *h*, respectively.

We can compare the calculated helicity density in the index matching case with the case of two different materials for the sub- and superstrates for the shifted structure (*d*_*y*_ = 100 nm). For this, we calculate the helicity density, , of the BIC eigenmode, i.e. with no incident field. Since there is no incident field, the helicity density is normalized to the maximum value of both cases for direct comparison. The results are shown in Figure 4. Figure 4a shows a perfectly balanced helicity density due to symmetry which integrates to zero over the whole space. Conversely, Figure 4b shows a greater contribution of the positive values of the helicity and results in a nonzero (positive) total helicity of the field distribution when integrated. For a more quantitative comparison, the color bar top and bottom numbers display the maximum and minimum values of *h*, respectively. Finally, it is worth noting that the local positive values of the helicity density are located on the superstrate (which could play the role of an active material), while the vast majority of the negative values lie on the quartz substrate (optically inactive). This yields an even higher helicity for the superstrate, which is especially relevant for an experimental configuration with an active material.

### 3.3 Helicity Density and Near Field

**Figure 5:**
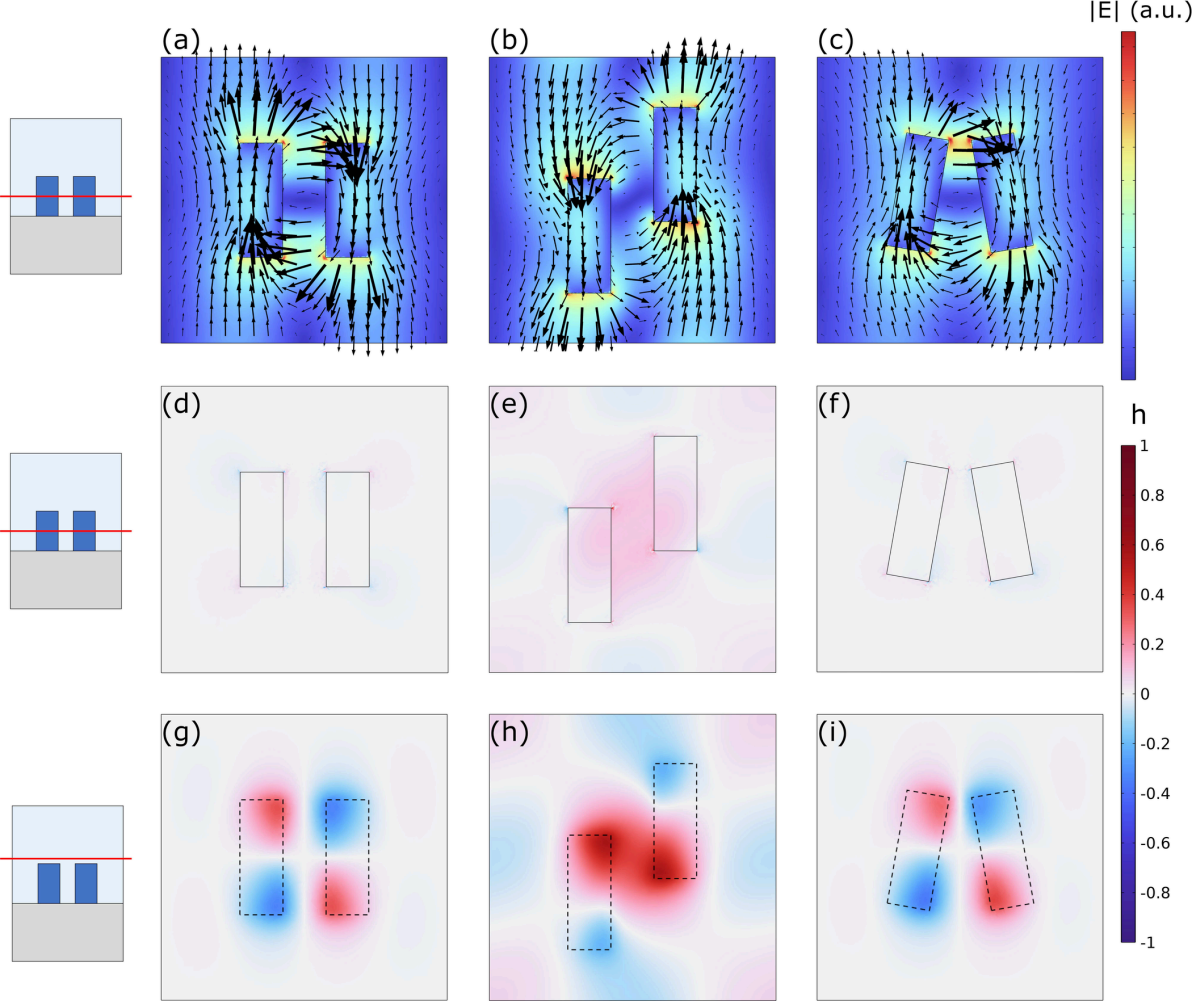
Near-field COMSOL calculations for the *p*_*y*_ BIC eigenmode (with no incident field) for Si rod dimer metasurfaces with rod and lattice dimensions as in Figure 2. (a–c) NF distribution of the electric field on the *xy*-plane crossing the center of the rods; the length of the arrows is proportional to the electric field magnitude. (d–f) Normalized helicity density on the *xy*-plane crossing the center of the rods. (g–i) Normalized helicity density on the *xy*-plane 15 nm above the top surface of the rods. (a, d, g) *d*_*y*_ = 0 nm; (b, e, h) *d*_*y*_ = 100 nm; (c, f, i) φ = 10°.

BICs are renowned for being completely decoupled from the radiative continuum, which makes them unable to be excited from the FF. Thus, in order to study the NF distribution of these modes we must study the eigenmode obtained as a solution of the Maxwell’s equations within the MS. We numerically calculate the eigenmode associated with the *p*_*y*_ BIC for this system in the three previous configurations: neutral, shifted, and slanted. The results are presented in Figure 5. Panels (a-c) show the NF distribution of the electric field on the *xy*-plane crossing the center of the rods, the length of the arrows is proportional to the electric field magnitude. As expected, the electric field inside each rod presents opposite orientations mostly aligned along the *y* axis, in agreement with the upper schemes of Figure 2. The antisymmetry results in the absence of radiation at the Γ-point, giving rise to the BIC for the neutral and shifted configurations. Panels (d-i) present the calculated helicity density normalized to the maximum value of the three cases for direct comparison. Figures 5d–f show the helicity density on the *xy*-plane crossing the center of the rods, while Figures 5g–i show the helicity density on the *xy*-plane 15 nm above the rods top surface (see lateral schemes). For all cases, the helicity density is much higher in the region above the rods, than in the space between them. The neutral case, Figure 5g, presents a perfectly symmetrical distribution of the helicity density that integrates to zero, which lets us conclude that this case is not intrinsically chiral, as expected. Something similar occurs with the slanted case, Figure 5i. The helicity density distribution is only slightly modified compared to the neutral case and the integral is still zero. This proves that the slanted configuration is intrinsically nonchiral in the NF in agreement with its *yz* mirror symmetry, even if it shows a strong off-normal CD in the FF. Finally, the shifted case, Figures 5e and h, presents a nonzero (positive) total helicity that is not compensated by symmetry. If compared to the neutral case, Figure 5g, the *y*-displacement approximates the two regions with positive values of the helicity, i.e. top region of the left rod and the bottom region of the right rod. This effectively enhances the local helicity density that can also be observed in the region between the rods, Figure 5e. The discussion is analogous for a negative value of *d*_*y*_, but in that case, resulting in an enhancement of the negative helicity.

**Figure 6:**
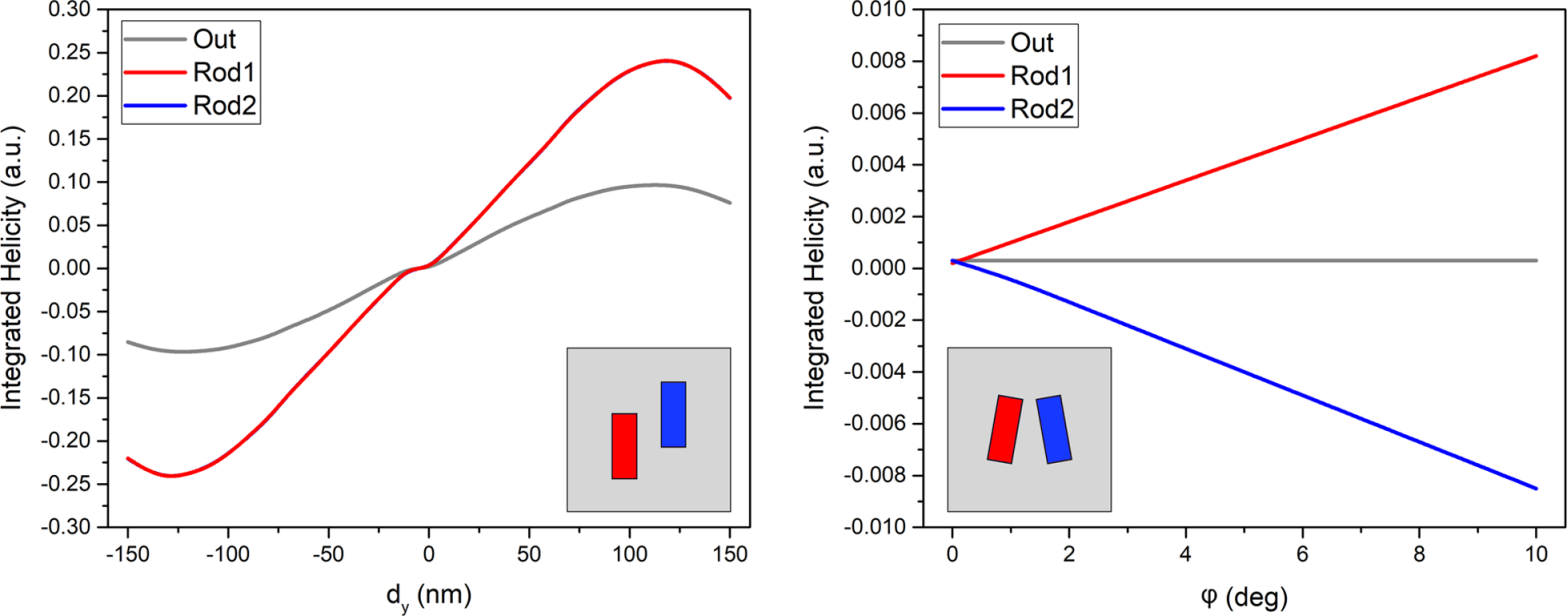
Integrated helicity density and its dependence on the asymmetry parameters for Si rod dimer metasurfaces with rod and lattice dimensions as in Figure 2.

In order to have a more quantitative view of the intrinsic chirality of the systems we computed the total helicity by integrating the helicity density in three relevant regions of the system: the left rod (Rod1), the right rod (Rod2), and a layer 200 nm in thickness surrounding the rods (Out). The results are presented in Figure 6 for the shifted and slanted cases as a function of the relevant asymmetry parameter in each case: vertical displacement (*d*_*y*_) for the shifted and rotation angle (φ) for the slanted. The results are radically different. The shifted system presents a positive total helicity for the three regions, Rod1, Rod2, and Out, when *d*_*y*_ > 0 in agreement with the results of Figure 5. The helicity presents the opposite behavior when *d*_*y*_ < 0, as expected. Note that the curves for Rod1 and Rod2 perfectly overlap, resembling the *C*_2_ symmetry of the whole system. We can conclude that this configuration presents a net intrinsic chirality in the NF which can be tuned, including its sign, by varying *d*_*y*_. Conversely, the slanted case shows opposite helicities for Rod1 and Rod2 that sum up to zero, with a constantly negligible helicity in the Out region. As a result, the total helicity of this configuration is always zero which results in the absence of intrinsic chirality.

**Figure 7:**
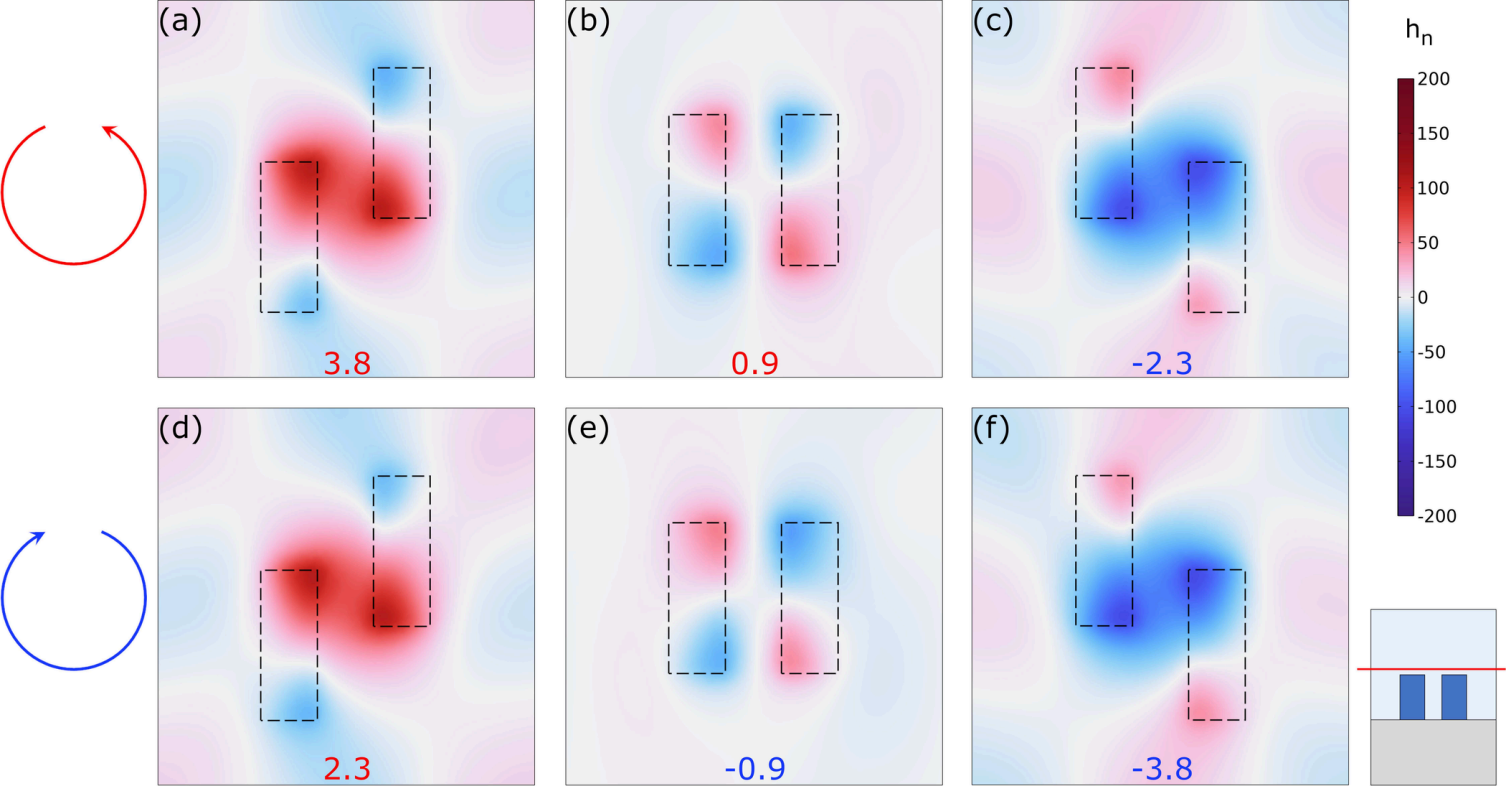
Induced helicity density under FF CPL illumination at θ = 1° on the *xy*-plane 15 nm above the nanorods for Si rod dimer metasurfaces with rod and lattice dimensions as in Figure 2. The helicity density is normalized to the helicity density of the incident plane wave: *h*_n_ = *h*/*h*_CPL_. The colored numbers represent the integrated and normalized helicity in a 200 nm layer surrounding the rods (Out). (a, d) *d*_*y*_ = 100 nm, (b, e) *d*_*y*_ = 0 nm, (c, f) *d*_*y*_ = −100 nm.

Finally, to unify both pictures, FF and NF, we analyzed the locally induced helicity when illuminated by FF plane wave excitation. To do so, we need an incidence θ ≠ 0, since the exact BIC at normal incidence will be decoupled from the radiative continuum. We chose θ = 1°, for which the state becomes a q-BIC with a finite but high Q factor. We illuminate the system with a circularly polarized plane wave matching the quasi-BIC frequency obtained from the extinction bands and analyze the induced helicity density in the structure. The results are presented in Figure 7 for RCP (a-c) and LCP (d-f) light. The three cases correspond to *d*_*y*_ = 100, 0, and −100 nm, respectively, and the helicity density is normalized to the helicity density of the incident plane wave (eq 4): *h*_*n*_ = *h*/*h*_*CPL*_. The colored numbers represent the integrated helicity in the superstrate (200 nm layer). The first thing to be noted is the extremely high local values obtained for the normalized helicity density, which reach values up to ±183 in this case, corresponding to a highly superchiral electromagnetic field. If we look at the neutral case, Figures 7b and e, the absence of chirality results in an integrated helicity close to ±1 in the Out region, corresponding to the helicity of the incident field. On the other hand, the shifted case appears to be highly independent of the incident field handedness. In Figures 7a and d, the induced total helicity is positive for both RCP and LCP illumination, in agreement with the intrinsic helicity of the BIC eigenmode, see Figure 5h. It is worth noting that the helicity in the Out region is almost double for LCP than RCP illumination. This manifests a much higher coupling of the FF and NF when the incident field helicity matches the intrinsic chirality of the structure. Similar conclusions can be obtained for the negatively shifted case (*d*_*y*_ = −100 nm), however, in this case, the intrinsic chirality (and the helicity) takes negative values, and the NF shows a stronger coupling with the RCP light, as showed in Figures 7c and f.

Interestingly, bear in mind that the helicity density is of paramount importance in processes involving chiral emission near/at the metasurfaces. The degree of circular polarization (DCP) of the light emitted by thin films of achiral molecules or quantum dots coating the metasurface, such as in the experimental works in refs (68−71), will be directly connected to the integrated helicity density. Thus, we include in Table 1 the integrated and normalized helicity in films of various thicknesses coating the nanorod dimer arrays shown in Figure 7 to shed light on the expected DCP. Recall that the helicity density is larger near/inside the Si rods, so the integrated helicity is larger for a film thickness coating only the region close to the nanorods, see Figure 4, whereas films significantly thicker or thinner than the nanorods height yield lower integrated helicity density due to the normalization (roughly speaking, the contribution from achiral emitters located in regions with lower helicity density increases, decreasing the DCP). According to this, we can see in Table 1 that the maximum integrated helicity is observed for a 200 nm layer, which is a layer thick enough to include the highest values of the helicity, but not excessively thick. Interestingly, note the remarkable differences inside each rod in the shifted case even though the (extrinsic) symmetry is merely broken by 1° incidence; the strong resonant behavior of the q-BIC with a huge *Q*-factor may be enhancing this helicity asymmetry. Recall also, that in a real experiment, the system should be solved for every different thickness and thus the NF distribution will be modified.

**Table 1:** Integrated and Normalized Helicity on the Different Regions Represented in Figure 7 for θ = 1° and for Different Thicknesses of the Outer Region: 100, 200, and 300 nm

System	Polarization	Rod1	Rod2	Out (100 nm)	Out (200 nm)	Out (300 nm)
Neutral (*d*_*y*_ = 0 nm)	L (+)	–5.99	8.27	0.86	0.90	0.84
	R (−)	5.99	–8.27	–0.86	–0.90	–0.84
Shifted (*d*_*y*_ = 100 nm)	L (+)	–0.01	13.56	3.61	3.83	2.83
	R (−)	10.38	1.04	2.14	2.26	1.31

## 4 Conclusions

We presented an analysis of the chiral behavior of bound states in the continuum (BICs) supported by metasurfaces based on poly-Si rod pairs in the visible range. We analyzed the circular dichroism (CD) of the far-field (FF) interaction, as well as the helicity of the near-field (NF) distribution for different configurations. We show that to achieve high values of CD it is best to use a system that allows the mixing of the orthogonal components of the fields, which hinders the attainment of a pure BIC and typically results in a quasi-BIC (q-BIC), as shown in the slanted structure. This will be the best approach to achieve chiral response in a FF configuration based on extrinsic chirality, like reflection or transmission experiments. On the other hand, the highest values of the helicity density are achieved for the *C*_2_ symmetric system with no mirror planes, as it presents intrinsic chirality. This is the desired situation when dealing with NF interactions such as strong-coupling, photoluminescence emission, or other local light-matter interactions. The values of the helicity density are shown to surpass the helicity density of circularly polarized plane waves by more than 2 orders of magnitude. This shows the possibility of obtaining superchiral electromagnetic fields in this kind of system while preserving the properties of BICs. Finally, the breaking of the *z*-axis symmetry is shown to be fundamental to achieving local intrinsic chirality. This can be easily obtained by selecting substrates and superstrates with different optical properties, i.e. refractive index, without losing the conditions that allow the emergence of BICs.

These results shed light on the possibility of manipulating and enhancing the chirality of electromagnetic fields at the nanoscale by using BICs. This can be accomplished both on the FF regime, with applications on filters, polarizers, etc., and the NF regime, enhancing chiral light-matter interaction like chiral photoluminescence emission or even chiral lasing. This will allow us to take advantage of the unique properties of BICs and q-BICs, such as extremely high Q factors and enhanced light-matter interaction, together with the large helicity densities that can be achieved in the NF of superchiral light, as demonstrated in this work.

## Supporting Information Available

S1. Multipolar decomposition of the rod dimers and FDTD details. S2. Angular dispersion of the extinction for TE and TM polarizations calculated with CEMD. S3. Details of the COMSOL simulations (PDF)
