# Supplementary material for: Superchiral Light Emerging from Bound States in the Continuum in Metasurfaces of Si Nanorod Dimers
Source: ACS Photonics. 2024 Sep 3;11(10):4090–100. doi: 10.1021/acsphotonics.4c00938 (PMC11488143; doi:10.1021/acsphotonics.4c00938)
Supplement: Supplementary file 1 — ph4c00938_si_001.pdf [file ph4c00938_si_001.pdf]

Superchiral light emerging from Bound States in the Continuum in metasurfaces of Si nanorod dimers

Jose Luis Pura,[*,†,‡] Beatriz Castillo López de Larrinzar,[¶] Minpeng Liang,[§,‖] Antonio García-Martín,[¶] Jaime Gómez-Rivas,[§,‖] and José A. Sánchez-Gil[†]

†*Instituto de Estructura de la Materia (IEM), Consejo Superior de Investigaciones Científicas, Serrano 121, 28006 Madrid, Spain.*

‡*GdS-Optronlab, Física de la Materia Condensada, Universidad de Valladolid, Paseo de Belén 19, 47011 Valladolid, Spain.*

¶*Instituto de Micro y Nanotecnología IMN-CNM, CSIC, CEI UAM+CSIC, Isaac Newton 8, Tres Cantos, 28760 Madrid, Spain.*

§*Department of Applied Physics and Science Education and Eindhoven Hendrik Casimir Institute, Eindhoven University of Technology, P.O. Box 513, 5600 MB Eindhoven, The Netherlands.*

‖*Institute for Complex Molecular Systems-ICMS, Eindhoven University of Technology, P.O. Box 513, 5612 AJ, Eindhoven, The Netherlands.*

E-mail: joseluis.pura@uva.es

S1. Multipoles

The finite differences in time domain (FDTD) simulation has been carried out using the commercial software Lumerical®, considering the Poly-Si rods immersed in a homogeneous medium with a refractive index of n = 1.46. Due to the an-isotropic nature of the rods, it is necessary to obtain the polarizabilities for different incidences and polarizations. The simulation time was established to last 10 ps, which allows for the wave to have exited the simulation region. The size of the simulation region is (1.3x1.3x8)μm, with perfect matching layers as boundary conditions. The FDTD requires meshing the whole space inside the simulation cell, the meshing details are δx = 0.0015 μm, δy = 0.002 μm, δz = 0.0015 μm in a region surrounding the rod, and increasing to reach a maximum of 0.1μm close to the boundaries of the simulation cell. Then by using the methodology in[1,2] we obtain the necessary multipoles $(\mathbf{p}, \mathbf{m})$ from which we extract the required polarizabilities for the CEMD as (see Fig. S1):

$$\begin{pmatrix} \mathbf{p} \\ \mathbf{m} \end{pmatrix} = \begin{pmatrix} \tilde{\alpha}_e & 0 \\ 0 & \tilde{\alpha}_m \end{pmatrix} \begin{pmatrix} \mathbf{E}_{inc} \\ \mathbf{H}_{inc} \end{pmatrix} \tag{1}$$

with

$$\tilde{\alpha}_e = \begin{pmatrix} \alpha_{e,x} & 0 & 0 \\ 0 & \alpha_{e,y} & 0 \\ 0 & 0 & \alpha_{e,z} \end{pmatrix} \text{ and } \tilde{\alpha}_m = \begin{pmatrix} \alpha_{m,x} & 0 & 0 \\ 0 & \alpha_{m,y} & 0 \\ 0 & 0 & \alpha_{m,z} \end{pmatrix} \tag{2}$$

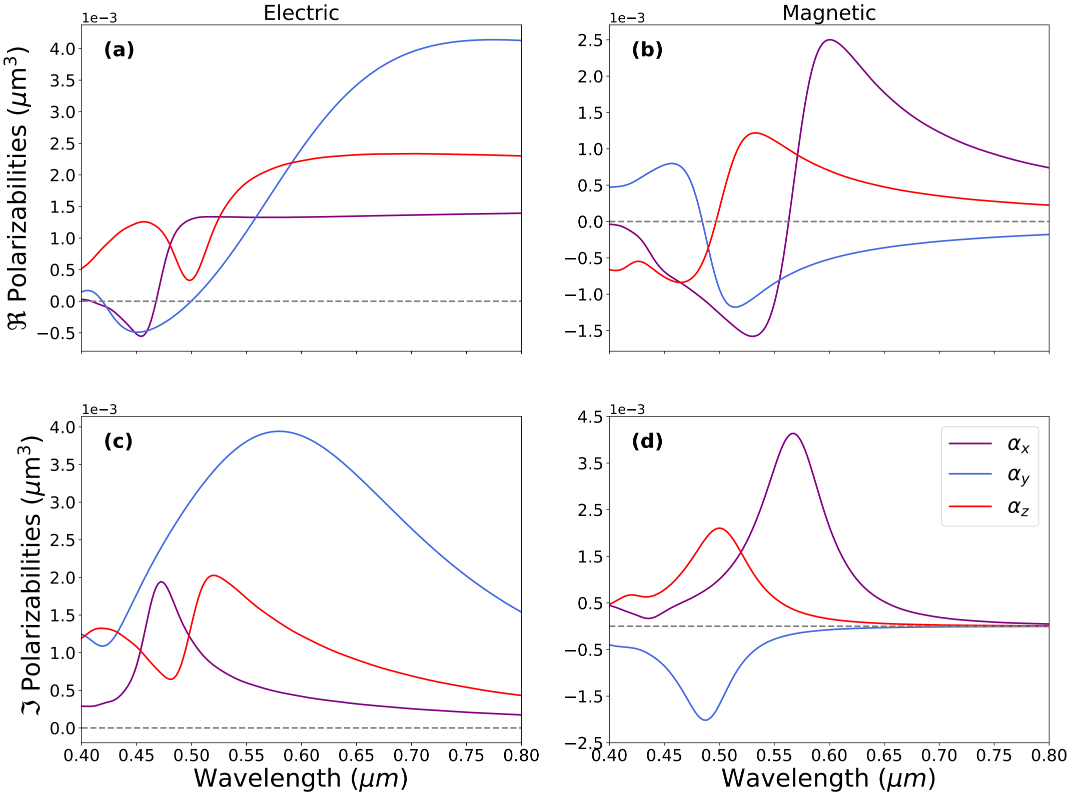

Figure S1: Real part of the electric (a) and magnetic (b) polarizabilities used in the CEMD. (c) and (d) contain the imaginary part of the electric and magnetic components respectively.

S2. Angular Dispersion

Figure S2 shows the angular dispersion of the optical extinction for TE and TM polarizations and for the three studied cases: neutral, shifted, and slanted. Figure S3 shows the extinction bands (TE polarization) calculated with the full CEMD and including only the polarizability of the y-direction electric dipole, $\alpha_{e,y}$.

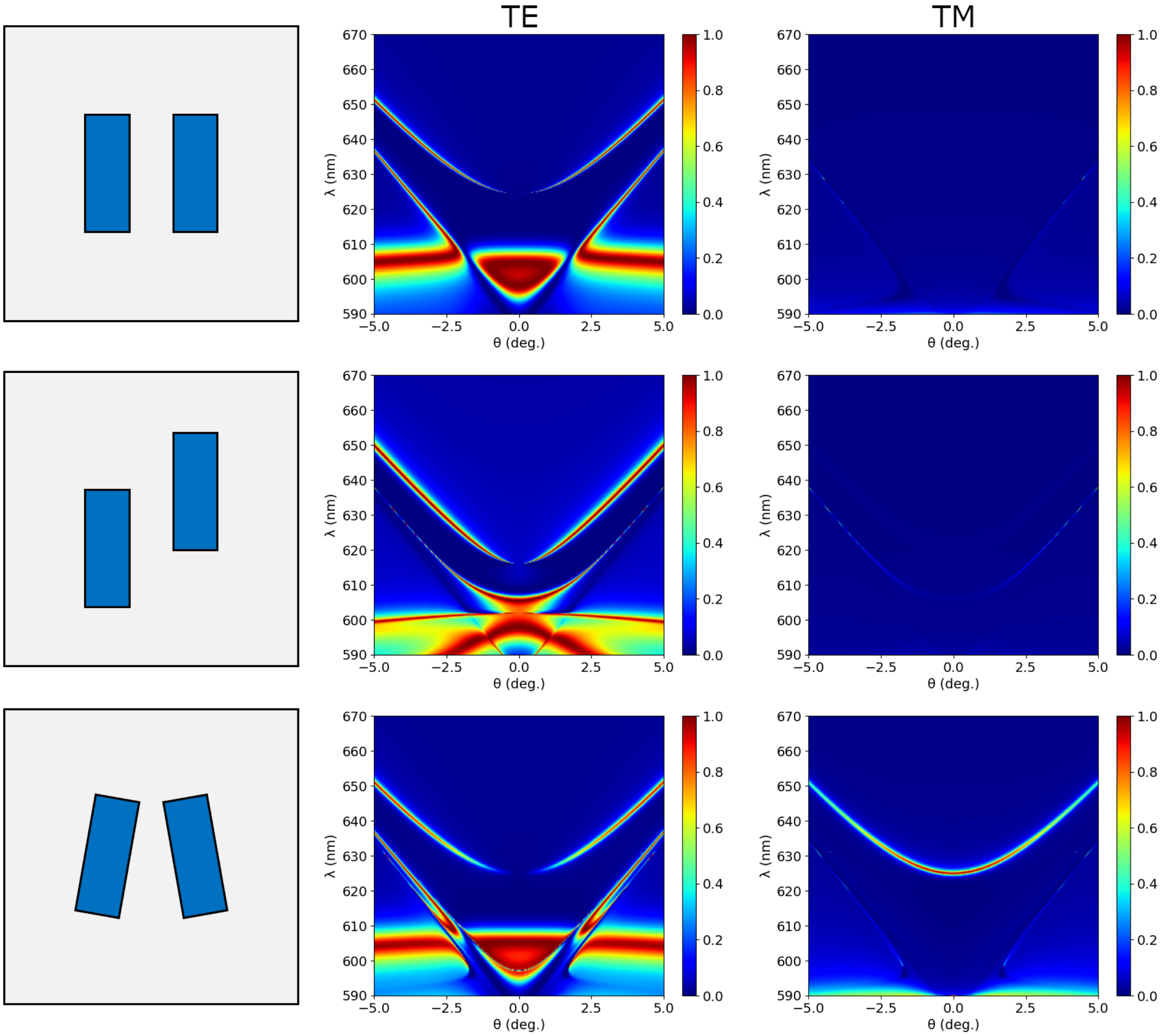

Figure S2: TE and TM bands calculated with CEMD for the three studied cases: neutral, shifted, and slanted. Note that a TM band only appears for the slanted case.

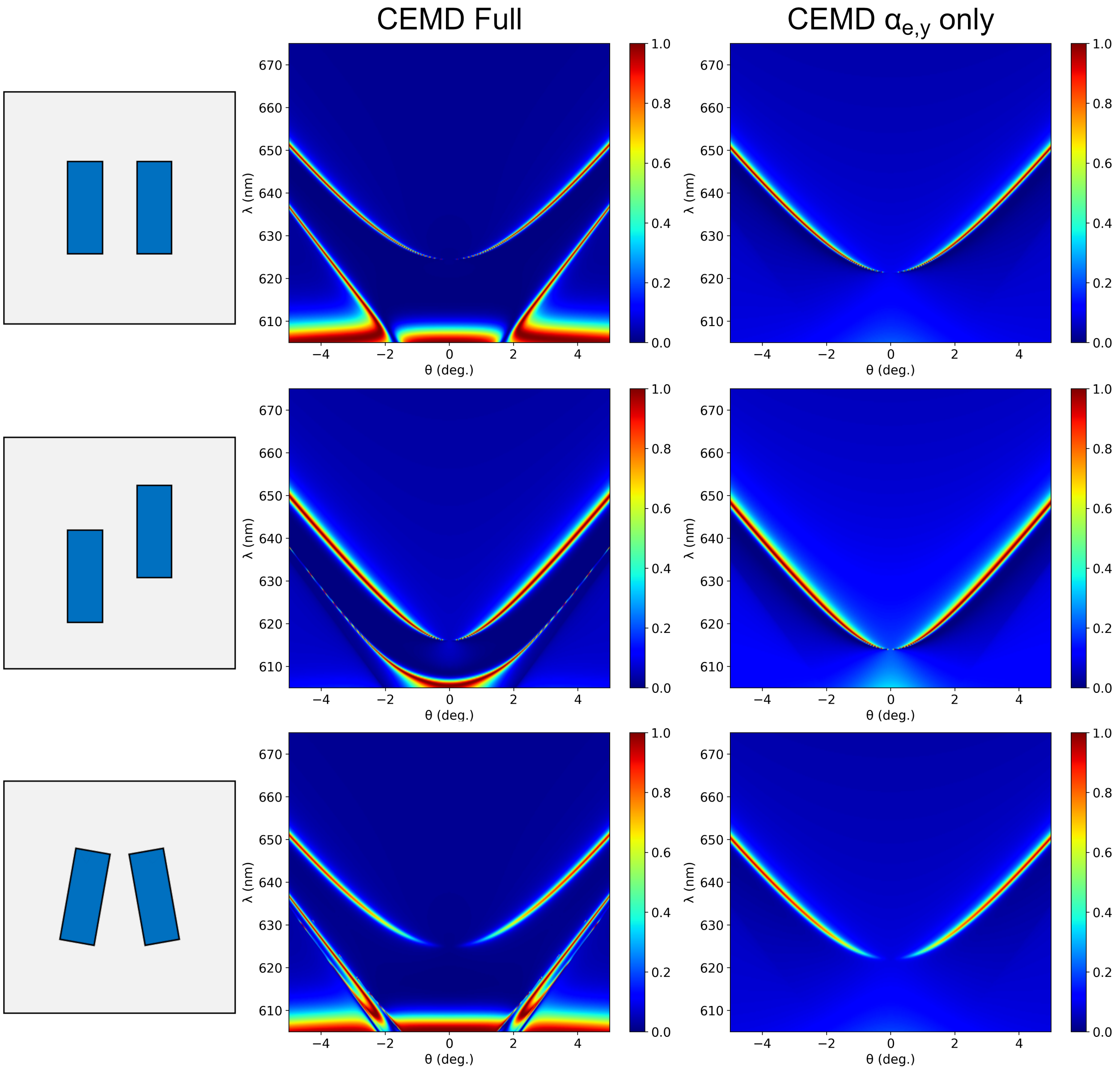

Figure S3: Extinction bands (TE polarization) calculated with the full CEMD and including only the polarizability of the y-direction electric dipole, $\alpha_{e,y}$.

S3. COMSOL Multiphysics

The finite element methods (FEM) simulation has been carried out using the commercial software COMSOL Multiphysics, considering the Poly-Si rods immersed in a homogeneous

medium with a refractive index of n = 1.46, and the rods lying on a substrate with n = 1.46, and a superstrate with n = 1.6 on the upper half-space. The size of the simulation region is $0.4 \times 0.4 \times 8.8\ \mu m^3$, with perfectly matching layers as boundary conditions in the $\hat{z}$-direction, and periodic boundary conditions on the $\hat{x}$ and $\hat{y}$-directions. The mesh inside the rods is set to a maximum element size of $\lambda_{min}/45$ while the substrate and superstrate regions are set to a maximum element size of $\lambda_{min}/15$. The system is solved under scattering conditions (frequency domain) to calculate the extinction bands and the CD. Also, the system is solved in the absence of external radiation to calculate the eigenmodes of the MS.

References

(1) Alaee, R.; Rockstuhl, C.; Fernandez-Corbaton, I. An electromagnetic multipole expansion beyond the long-wavelength approximation. *Optics Communications* **2018**, *407*, 17–21.

(2) Castillo López de Larrinzar, B.; Lanzillotti-Kimura, N. D.; García-Martín, A. Interaction effects in chiral acoustoplasmonic nanostructures. *Proceedings of SPIE, Nanophotonics X* **2024**, *12991*, 129910B.
